# A double-blind, randomized pilot study for comparison of *Melissa officinalis* L. and *Lavandula angustifolia* Mill. with Fluoxetine for the treatment of depression

**DOI:** 10.1186/s12906-020-03003-5

**Published:** 2020-07-03

**Authors:** Mostafa Araj-Khodaei, Ahmad Ali Noorbala, Reza Yarani, Fatemeh Emadi, Elham Emaratkar, Soghrat Faghihzadeh, Zahra Parsian, Fatemeh Alijaniha, Mohammad Kamalinejad, Mohsen Naseri

**Affiliations:** 1 Department of Traditional Medicine, School of Medicine, Shahed University, 1471, North Kargar, Engelab Square, Tehran, Iran; 2 Aging Research Institute, Tabriz University of Medical Sciences, Tabriz, Iran; 3 Department of Persian Medicine, School of Traditional Medicine, Tabriz University of Medical Sciences, Tabriz, Iran; 4 Psychosomatic Medicine Research center, Psychosomatic Ward, Imam Khomeini Hospital, Tehran University of Medical Sciences, End of Keshavarz Blv, Tehran, Iran; 5 Department of Pediatrics E, Copenhagen Diabetes Research Center (CPH-DIRECT), Herlev University Hospital, Herlev, 2730 Copenhagen, Denmark; 6 Medical Biology Research Center, Kermanshah University of Medical Sciences, Kermanshah, Iran; 7 Traditional Medicine Clinical Trial Research Center, Shahed University, 1471, North Kargar, Engelab Square, Tehran, Iran; 8 Department of Biostatistic and Epidemiology, School of Medicine, Zanjan University of Medical Sciences, Mahdavi St., Karmandan Town, Zanjan, Iran; 9 Emergency Medicine Research Team, Daneshgah St. Imam Reza Hospital, Tabriz University of Medical Sciences, Tabriz, Iran; 10 School of Pharmacy, Shahid Beheshti University of Medical Sciences, Tehran, Iran

**Keywords:** Depression, Traditional Persian medicine, Herbal medicine, Fluoxetine, *Melissa officinalis*, *Lavandula angustifolia*

## Abstract

**Background:**

Depression has rapidly progressed worldwide, and the need for an efficient treatment with low side effect has risen. *Melissa officinalis* L and *Lavandula angustifolia* Mill have been traditionally used in Asia for the treatment of depression. Many textbooks of traditional Persian medicine refer to these herbs for the treatment of depression while there are no adequate clinical trials to support this claim.

The present study aimed to evaluate the efficacy of *M. officinalis* and *L. angustifolia* compared to fluoxetine for the treatment of mild to moderate depression in an 8-week randomized, double-blind clinical trial.

**Methods:**

Forty-five adult outpatients who met the Diagnostic and Statistical Manual of Mental Disorders, 5th edition (DSM-5) for major depression, were randomly assigned to 3 groups to daily receive either *M. officinalis* (2 g) or *L. angustifolia* (2 g) or fluoxetine (20 mg) and were assessed in weeks 0, 2, 4 and 8 by the Hamilton Rating Scale for Depression (HAM-D) including 17 items.

**Results:**

Our study showed that *M. officinalis* and *L. angustifolia* effect similar to fluoxetine in mild to moderate depression. (F = 0.131, df = 2,42, *p* = 0.877).

**Conclusion:**

Due to some restrictions in this study including absence of placebo group, large-scale trials are needed to investigate the anti-depressant effect of these two herbs with more details.

**Trial registration:**

IRCT2014061718126N1. Registration date: 2015-06-04-“Retrospectively registered”.

## Background

Depression as the multifactorial non-fatal disease has a high prevalence worldwide [1, 2] . It is estimated that in the coming next 20 years, depression will be the second cause of human disability [3]. Despite improvements in the treatment modalities, many patients experience recurrence relapses and side effects. The current chemical treatments have low efficiency with undesirable side effects, therefore, a need for new remedies to improve acute and long-term treatment outcomes, while minimizing the side effects is growing [4]. Today, many scientists employ traditional treatments to find the cure for the modern human diseases not only in clinical setting [5, 6], but also in basic research [7, 8]. Herbal remedies and complementary treatments which can overcome these shortcomings are therefore of high interest [9] ascribed to their low cost and minimal side effects with better patient compliance [10].

Documented medical experiences discussed in ancient sources can help us in deciding the treatments for the modern day diseases [5, 11, 12]. Therefore, reusing and/or finding new herbal treatments among the forgotten remedies for various diseases are now the focus of many studies [6, 13, 14]. Numerous documented sources, including pharmacological textbooks have been used for this purpose including Persian Medicine (PM). PM consists of extensive knowledge and practical experiences for disease treatment that have been in use from ancient times (more than 10,000 years ago) to the present. In PM, the emphasis is more on prevention rather than treatment. PM is mainly based on practical treatments and pure observation of the outcomes after treatments. In many cases, the patient’s behavior and reactions to the treatment have been documented in detail in old PM manuscripts. In PM, nutrition and remedies are the main treatments followed by manipulation [15–20].

In the current study, more than 10 authoritative pharmacological books of ancient medicine with a focus on antidepressants were searched, and drugs were prioritized based on their repetition and significance [21–23]. We used the reverse pharmacology method to find a smart approach for new drug candidates to simplify the discovery process [24]. Among these remedies, *M. officinalis* and *L. angustifolia* are the most significant ones. These remedies are cheap, affordable, indigenous and easily accessible [22, 23, 25].

*Melissa officinalis* L or Lemon Balm (Lamiaceae), contains volatile oil with citral and has a lemony aroma and taste. Historically, the use of lemon balm for therapeutic purposes dates back to “De Materia Medica” in about 50–80 B.C. [26]. In the middle ages, it was utilized in European countries as a medical agent based on Paracelsus’s recommendation [27]. It has been introduced as a revivifying ingredient and has been mentioned as an effective treatment for nervous system disturbances. New findings also indicate the spasmolytic, anti-bacterial and behavioral modulator properties of lemon balm [28, 29]. It is believed that lemon balm helps the sleep disturbance, resulted from its sedative effect. It is effective in reducing the nervous system disorders, since it reduces excitability, anxiety, and stress [30]. Avicenna used *Melissa officinalis* as an exhilarating and anti-depressant medication 1000 years ago [31]. Animal studies evaluated the anti-depressant effect of *Melissa officinalis*, through inhibition of MAO (Monoamine Oxidase) or enhancement of norepinephrine neurotransmissions [32, 33]. Furthermore, *Melissa officinalis* contains flavonoids, phenolic acid and tannins [34]. The famous remedy, St. john’s effects as anti-depressant through flavonoids [35]. Although the evidence for the presence of flavonoids in *Melissa officinalis* as well as animal studies [36] have proved the anti-depressant effective of this herb, there is no clinical trial evaluating its anti-depression effects in patients.

*Lavandula angustifolia* Mill or Lavender (Lamiaceae) is an aromatic and evergreen subshrub that natively grows in the Mediterranean [37]. It is used for mood disturbance such as restlessness or insomnia, nervous stomach irritation and nervous intestinal discomfort [30]. There are limited data regarding the effect of Lavender on depression. However, it has been shown to increase the strength of the nervous system, and it seems to be effective in reduction of depression and nervous exhaustion [38, 39]. In one of the two only available human studies conducted in 2002, it was indicated that lavender could be an effective adjuvant therapy for depression [40]. In addition, Chen et al. in 2015, showed that lavender tea could have a short immediate effect on alleviation of postpartum depression, but long-term effect was not demonstrated [41]. Despite Avicenna’s opinion about the efficiency of lavender in the treatment of depression and presence of flavonoids in the remedy, there is not sufficient clinical evidence to prove lavender‘s effectiveness. Therefore, the need for further investigation is obvious.

Our objective in this study was to compare the efficacy of lemon balm and lavender to fluoxetine in the treatment of mild to moderate depression in an 8-week double blind randomized trial. The findings from this study can lead to treatments with higher efficacy and fewer side effects, as well as lower cost for the patient with higher compliance.

## Methods

### The study

The study was an 8-week double blind randomized clinical trial conducted in Imam Khomeini hospital in Tehran, Iran from October 2014 to July 2015. The research protocol was approved by the regional ethic committee of Shahed University (No.41/226696), and all the patients were informed about the content and procedure of the experiment and presented the written informed consent. The study also was conducted in accordance with Helsinki’ declaration and its subsequent revisions. This project was registered at the Iranian Clinical Trials Registry (IRCT2014061718126N1; www.irct.ir).

### Patient selection

A total of 45 adult patients participated in this study. Outpatients who met the criteria for the Diagnostic and Statistical Manual of Mental Disorder, Fifth Edition (DSM-V) (American psychiatry Association, 1994) were evaluated for medical history and other health issues. Inclusion criteria were as follows: patients who met the Diagnostic and Statistical Manual of Mental Disorder, (DSM-5) (American psychiatry Association) for mild to moderate depression; patients who had a baseline Hamilton Rating Scale for Depression (HAM-D 17-item) scores between 8 and 24; age between 18 and 65 and inform consent. Exclusion criteria were also as follows: serious chronic disease, life threatening illness, thyroid disease; psychosis and other psychiatric disorders based on the DSM-V axis I or II evaluated by structured diagnostic interview; suicide history; pregnancy and lactation; history of sensitivity to fluoxetine or herbal compounds; consumption of alcohol or other addictive agents during last 2 weeks; consumption of psychotropic medication, alternative medicine or psychotherapy for at least 4 weeks before the study entry.

As depression is one of the first and the most important risk factors of suicide, patients who had a significant risk of suicide (score > or = 2 on the suicide item of HDRS) at any time during participation were dropped out. Our psychiatrist, who evaluated the patient’s conditions, excluded these patients in any time of study, and subsequent treatment was performed for them. Furthermore, the patients whose depression severity changed to severe depression (HAM-D > 24) or had severe drug side effects (including any hypersensitive reaction, severe anxiety, moderate to severe nervousness, severe itching and fatigue); were excluded from the study before completion, and were referred to psychiatrists for routine treatment.

### Treatment medications

#### Herbal drugs

The dosages of herbal treatments.

### Lemon balm

Dried *L. angostifulia* and *M.officinalis* were purchased from market and voucher specimen (number PMP-325 and PMP- 410 respectively for *L. angustifolia* and *M. officinalis*) was deposited at the Herbarium of Faculty of Pharmacy, Tehran University of Medical Sciences, Tehran, Iran. Dried leaves of lemon balm were used to prepare capsules. For this purpose, additional parts of plants were removed, and the leaves were cleaned. The leaves were completely powdered by a grinder. The powder was kept away from light and moisture before and after preparation. The powders kept in capsules were filled with 500 mg prepared powder.

### Lavender plant

Lavender plant with the scientific name of *L. angostifulia* is also prepared with the same procedure as lemon balm and used with at 500 mg dose.

#### Fluoxetine

Fluoxetine was provided as powder from Dr. Abidi’s Pharmaceutical Company. Fluoxetine powder was filled in capsules, which were exactly similar to the Melissa and Lavender capsules in terms of size and color. However, each capsule of fluoxetine contained 5 mg fluoxetine powder and 495 mg starch powder.

### Assay of herbal drugs

Total contents of phenolic and flavonoid compounds of the both plants were determined using the spectrophotometric method [42, 43]. The contents of phenolic compounds using Folin-Ciocalteu’s reagent and gallic acid as a standard for *M. officinalis* and *L. angostifulia* were 4.88 ± 0.025 mg GA/g and 5.04 ± 0.018 mg GA/g, respectively. The contents of flavonoids in the *M.officinalis* and *L. angostifulia* using rutin as a standard, was determined 4.28 ± 0.006 and 5.32 mg ± 0.001 RU/g, respectively.

### Study design and intervention

All the patients underwent a standard clinical assessment, including a psychiatric examination, the medical history and a diagnostic psychiatric interview. The patients were randomly assigned to receive two capsules (1 g) of lemon balm (group 1) or lavender (group 2) every 12 h (1 g/BD) or two capsules of fluoxetine (10 mg) (group 3) every 12 h for 8 weeks. Grouping of the patients was randomly performed as follows: the first patient was placed in the first group, and the second patient in the second group and the third patient in the third group, and the rest of the patients were grouped in the same order. Gender was randomly separated between the groups with no preference. Capsules were packed in a container and were identifiable by a private code on the container, which was coded by a third party who had no role in the study. These codes were kept as a secret until the end of data analysis. Eligible patients were assessed primarily by a physician (PhD student) and then confirmed by a psychiatrist in baseline and 2, 4 and 8 weeks after therapy. In our study, the main part of outcome was the 17-item HAM-D assessing the severity of depression. As this was a pilot study and the first evaluation of *Melissa officinalis* as well as owing to ethical considerations, only patients with mild to moderate depression were selected, and patients who had severe depression and their score of Hamilton depression was more than 24, were excluded from the study. None of the researcher, psychiatrist, data analyzer and patients was aware of the medication types and concentration (all medication information was confidential) throughout the survey.

### Side effects

A checklist of probable side effects of drugs used in this study was given to all the patients. This checklist was completed by the physician in weeks 2, 4 and 8. Patients could have phone consultation with the physician during the survey.

### Statistical analysis

A two-way repeated measures analysis of variance (time– treatment interaction) was used in the study. Three groups as between subjects factors (group) and the four two weekly measurements during treatment as the within-subjects factor (time) were considered. This was carried out for HAM-D total scores. To compare the three groups at baseline, and the outcome of three groups at the end of the trial, one-way ANOVA and if necessary Kruskal-Wallis test were used. Normality of data was evaluated using the Kolmogorov-Smirnov test. Results were presented as mean ± SD. Differences with *P* < 0.05 were considered significant.

## Results

### Demographic characteristics

**Fig. 1:**
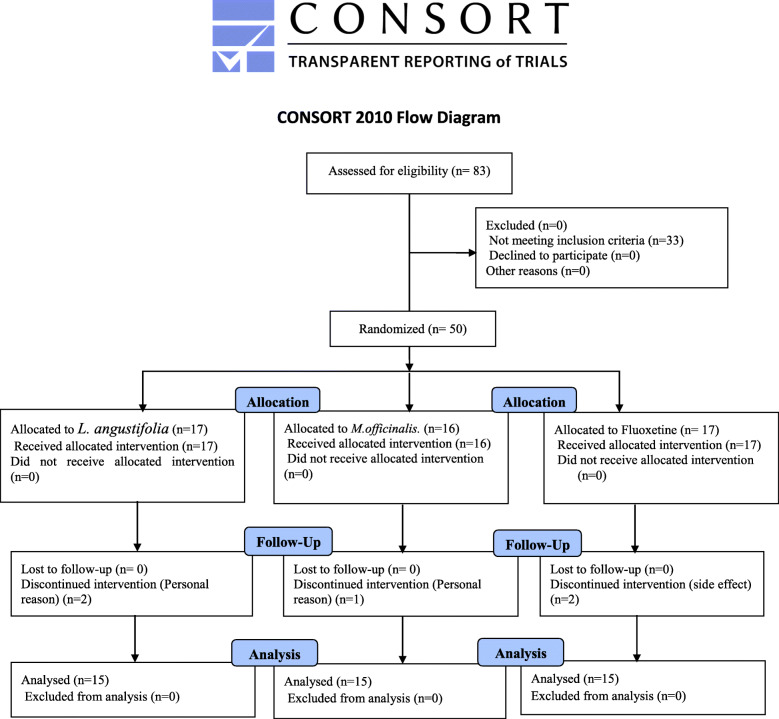
Flow diagram of study participation

Eighty three patients were screened for eligibility criteria, and 50 patients were entered the study. These patients were divided into 3 groups so that groups 1, 2 and 3 had 17, 17 and 16 patients, respectively. (Group 1 received *L. angustifolia*, group 2 received fluoxetine, and group 3 received *M. officinalis*). Figure 1 shows the flow diagram. There were not significant differences in the baseline of the Hamilton Depression Rating Scale (F = 0.572, df = 2, *p* = 0.569), marriage (F = 0.556, df = 2, *p* = 0.757), and age (F = 0.722, df = 2, *p* = 0.492), among the groups. Table 1 briefly reports the characteristics of the three groups.

**Table 1:** Baseline information of the three groups

	*L. angustifolia*	Fluoxetine	*M. officinalis*	*P.Value*
Age (years, mean ± S	37.9 ± 2.4	33.4 ± 2.7	37.4 ± 3.3	*0.492*
Sex, n (%)				0.843
Women	11 (73.3)	11 (73.3)	10 (66.7)	
Men	4 (26.7)	4 (26.7)	5 (33.3)	
Marital status, n (%)				0.757
Married	9 (60)	9 (60)	10 (66.7)	
Single	6 (40)	7 (46.7)	5 (33.3)	
Baseline Hamilton Score	17.2 ± 3.61	18.4 ± 3.06	17.8 ± 3.04	0.569

### Retention in treatment

Forty-five patients (each group 15) completed the 8-week trial, while 2, 2, and 1 patient were dropped out of groups 1, 2, and 3, respectively, with their own consent except one patient in the fluoxetine group due to sexual dysfunction (Fig. 1). There was no significant difference between the groups in term of wrap up. (*P*-Value = 0.412).

### Effect on HAM-D scores

**Fig. 2:**
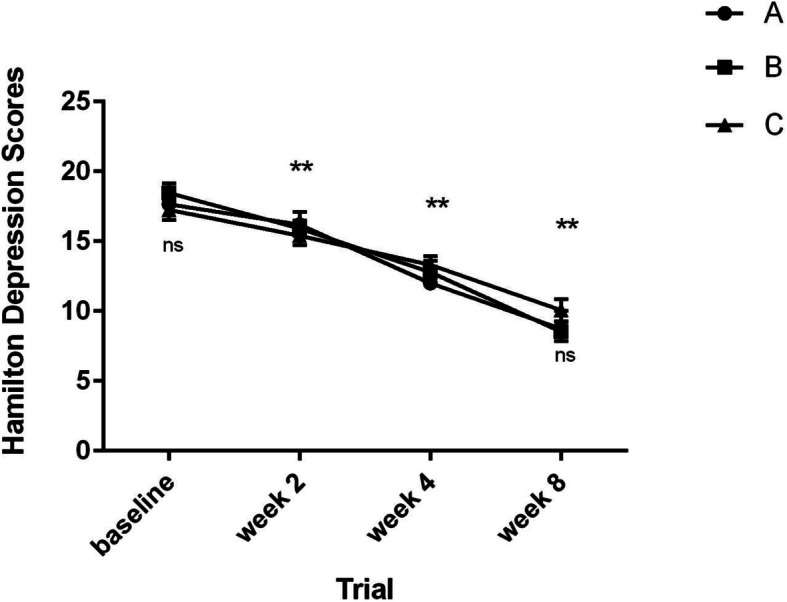
Mean + − S.E.M. scores of *Lavandula angustifolia* (Group A), Flouxetine (Group B) and *Melissa officinalis* (Group C) on the Hamilton Depression Rating Scale. The horizontal symbol (**) was used to express statistical significance versus their respective baseline value and the symbol ns (no significance) were used for between-group comparisons

Figure 2 shows the mean ± SEM scores of groups 1, 2 and 3. There was no significant difference in the depression baseline scores of these 3 groups from the start of the study (F = 0.572, df = 2,42, *p* = 0.569), and the same pattern was observed in week 8 (Chi^2^ = 0.330, df = 2,42, *p* = 0.848). Trend of changes was homogeneous for all the treatments during the evaluation period, and all the patients’ HAM-D score declined. Improvement of symptoms with all three treatments was statistically significant by week 8, while no marked difference was found between the groups. (F = 0.131, df = 2,42, *p* = 0.877). All the 3 groups showed the same pattern of performance throughout the study. In week 8, all the 3 groups showed a significant decrease in Hamilton score, While the interaction of groups and Hamilton score was not significant (*P* = 0.192), meaning none of the groups showed higher or lower pattern of decrease compared to the others. The changes at the end point compared to the baseline with 95% confidence interval for difference were: − 7.80, − 9.80, and − 8.46 for groups 1, 2 and 3, respectively.

### Side effects

There was no serious side effect or death from treatment. One patient in the fluoxetine group left the study due to diarrhea, and one of the patients receiving *L. angostifulia* suffered from drowsiness and left the group. Other adverse effects that patients experienced were trivial and resolved spontaneously in the course of treatment. Table 2 lists the adverse effects.

**Table 2:** Clinical complications and side effects were reported as number per group

Side effects	*L. angustifolia*	Fluoxetine	*M. officinalis*	*P*.Value
Anxiety	1(3 days)	5(3 days)	1(2 days)	0.179
Dizziness	1(1 day)	1(1 day)	2(1 day)	1
Dry mouth	2(2 days)	1(4 days)	1(5 days)	1
Decreased appetite	0	3(4 days)	1(2 days)	0.302
Headache	2(1 day)	4(1 day)	1(1 day)	0.463
Increased appetite	3(4 days)	1(3 day)	2(3 days)	0.858
Insomnia	0	3(3 days)	1(2 days)	0.302
Nausea	1(1 day)	0	1(1 day)	1
Sedation	2(3 days)	0	2(3 days)	0.524
Sexual dysfunction	0	3(7 days)	1(1 day)	0.302

## Discussion

This study showed that *Melissa officinalis* and *Lavandula angustifolia* had equal effect as Fluoxetine. As 13 million people are annually affected by depression, treating it can improve the people’s quality of life [44]. An ideal treatment should regulate the patient’s mood, and increase awareness, personal desires and affection, as well as reverse the functional and social disabilities associated with depression, along with decreasing the suicide rates [45].

Currently, there are few famous anti-depressant chemical pharmaceutics having some difficulties in application such as slow inception of act, poor remission rate and need for several continuous months of treatment for clinical improvement [46]. In the search for better and faster acting treatments with fewer side effects and higher patient’s compliance, we introduced two traditional herbal medications.

This randomized double-blind clinical trial is the first evaluation of efficacy of *M. officinalis* (lemon balm) and the fourth human study of *L. angustifolia* (lavender) compared to anti-depressants. Fluoxetine as a famous anti-depressant was used in this study. The findings of our study indicated that the *M. officinalis* and *L. angustifolia* was the same as fluoxetine in this study. There was no significant alteration in efficacy for alleviation of depression between the 3 groups of the study. Lemon balm and lavender were generally well tolerated with fewer side effects and lower drug attachment (addiction), and no sign of depression was observed after stopping the treatment in comparison to fluoxetine.

Our findings are in line with previously published studies that confirmed antidepressant effect of Lavender. One study by Akhondzadeh et al. (2002) consisted of 3 groups of patients with 15 individuals in each group who had mild to severe depression. The first group received lavender and placebo pills, the second group received imipramine and placebo pills, and finally the third group received both lavender and imipramine and no placebo pills in a 4-week period. The findings demonstrated that the combination of alcoholic extract of lavender and imipramine was more effective than sole imipramine [40]. In another interesting study, Kasper et al. (2016) demonstrated that Silexan, which is the active compound isolated from *L. angustifolia*, was significantly effective in reduction of depression compared to the placebo group. In this investigation, one placebo group (128 patients) and one treatment group (141 patients) of 269 participants were studied [47]. For 14 days, Chen evaluated the effect of drinking lavender tea on postnatal depression in women 6 weeks after giving birth. Lavender temporarily reduced symptoms of depression, but it did not last for a long time after intervention [41].

The anti-depressant effect of *M. officinalis* has also been demonstrated in few animal studies [48, 49]. In one study by Emamgoreishi et al., various doses of *M. officinalis* in forced swimming test were used. Their findings demonstrated the reduction in immobility and posed anti-depressant like activity similar to Imipramine [45]. Lin et al. (2015) showed that immobility duration was significantly reduced in acute and sub-acute phases in all doses of *M. officinalis*. Moreover, in swimming, duration increased only in the sub-acute phase. Serotonergic antidepressant-like activities of the aqueous extract of *M. officinalis* were approved in this study [46]. In the study by Taiwo et al. (2012), a significant decrease in immobility in male and female rats in 100 and 300 mg/kg doses of *M. officinalis* in the sub-acute phase in forced swimming test was observed [47]. Lin et al. (2015) and Taiwo et al. (2012) also reported similar results [46, 47]. Overall, our findings are in good agreement with these studies. The study by Solberg et al. is the only evaluation of *M. officinalis* in human depression. In this study they showed that combination of Young Tissue Extract (YTE) and *M. officinalis* as well as YTE alone could be effective in improving the depression scores on the HAM-D in comparison to placebo [50]. However, *M. officinalis* did not show any additional effect in reducing the Hamilton rating scale, which is in conflict with our study results. In the study conducted by Solberg et al., a comparison between the effects of the mentioned extracts to the common antidepressant medications as control is missing. Moreover, the effect of *M. officinalis* has not been studied solely; therefore, it is difficult to distinguish if the antidepressant effect was from *M. officinalis* or YTE, or the mixture.

In our study, the patients in the fluoxetine group experienced more insomnia, sexual dysfunction, anxiety and decreased appetite than those in the *lemon balm* and *lavender* groups*.* Instead*, lavender* and *lemon balm* had more sedative effects. However, frequencies of adverse effects were not significantly different.

The current study was a pilot study, and the interpretation of the outcomes must be considered with cautious, as the sample size was small and follow-up time was short. In some studies fluoxetine was ineffective in mild depression, but we didn’t consider a placebo group in our study to be compared with the treatment groups and this is a limitation for this trial [51]. Although in our study effects of *M. officinalis* and *L. angustifolia* was equal to fluoxetine, a meta-analysis has revealed that these effects are due to the placebo effect [52]. Regarding to this controversy, larger trials with longer duration of follow up and including placebo group are needed to evaluate the long-standing safety and efficacy of these herbal medications.

## Conclusion

According to the present study results, effects of *M. officinalis* and *L. angustifolia* are equal to fluoxetine, which is a well-known widely used anti-depression drug. However, due to the current controversy regarding its effectiveness in mild depression, comparing with a placebo group in larger clinical trials should be further evaluated. *M. officinalis* and *L. angustifolia* also showed fewer side effects compared to fluoxetine.

## Data Availability

The datasets generated and/or analyzed during the current study are not publicly available due to decision of author’s team but are available from the corresponding author on reasonable request.

## Abbreviations

TPM: Traditional Persian Medicine
DSM-V: Diagnostic and Statistical Manual of Mental Disorder, fifth edition
HAM-D: Hamilton Rating Scale for Depression
